# The Watts Connectedness Scale: a new scale for measuring a sense of connectedness to self, others, and world

**DOI:** 10.1007/s00213-022-06187-5

**Published:** 2022-08-08

**Authors:** Rosalind Watts, Hannes Kettner, Dana Geerts, Sam Gandy, Laura Kartner, Lea Mertens, Christopher Timmermann, Matthew M. Nour, Mendel Kaelen, David Nutt, Robin Carhart-Harris, Leor Roseman

**Affiliations:** 1 Centre for Psychedelic Research, Imperial College London, London, UK; 2 Acer Integration, London, UK; 3 Synthesis Institute, Amsterdam, Netherlands; 4 Department of Neuropsychology and Psychopharmacology, Faculty of Psychology and Neuroscience, Maastricht University, Maastricht, Netherlands; 5 Psychedelics Division, Department of Neurology, University of California San Francisco, Neuroscape, USA

**Keywords:** Psychedelics, Psilocybin, Alienation, Community, Transpersonal, Nature-connectedness, Relational, Depression, Transdiagnostic, Therapy

## Abstract

**Rationale:**

A general feeling of disconnection has been associated with mental and emotional suffering. Improvements to a sense of connectedness to self, others and the wider world have been reported by participants in clinical trials of psychedelic therapy. Such accounts have led us to a definition of the psychological construct of ‘connectedness’ as ‘a state of feeling connected to self, others and the wider world’. Existing tools for measuring connectedness have focused on particular aspects of connectedness, such as ‘social connectedness’ or ‘nature connectedness’, which we hypothesise to be different expressions of a common factor of connectedness. Here, we sought to develop a new scale to measure connectedness as a construct with these multiple domains. We hypothesised that (1) our scale would measure three separable subscale factors pertaining to a felt connection to ‘self’, ‘others’ and ‘world’ and (2) improvements in total and subscale WCS scores would correlate with improved mental health outcomes post psychedelic use.

**Objectives:**

To validate and test the ‘Watts Connectedness Scale’ (WCS).

**Methods:**

Psychometric validation of the WCS was carried out using data from three independent studies. Firstly, we pooled data from two prospective observational online survey studies. The WCS was completed before and after a planned psychedelic experience. The total sample of completers from the online surveys was *N* = 1226. Exploratory and confirmatory factor analysis were performed, and construct and criterion validity were tested. A third dataset was derived from a double-blind randomised controlled trial (RCT) comparing psilocybin-assisted therapy (*n* = 27) with 6 weeks of daily escitalopram (*n* = 25) for major depressive disorder (MDD), where the WCS was completed at baseline and at a 6-week primary endpoint.

**Results:**

As hypothesised, factor analysis of all WCS items revealed three main factors with good internal consistency. WCS showed good construct validity. Significant post-psychedelic increases were observed for total connectedness scores (η2 = 0.339, *p* < 0.0001), as well as on each of its subscales (*p* < 0.0001). Acute measures of ‘mystical experience’, ‘emotional breakthrough’, and ‘communitas’ correlated positively with post-psychedelic changes in connectedness (*r* = 0.42, *r* = 0.38, *r* = 0.42, respectively, *p* < 0.0001). In the RCT, psilocybin therapy was associated with greater increases in WCS scores compared with the escitalopram arm (*η*_*p*_*2* = 0.133, p = 0.009).

**Conclusions:**

The WCS is a new 3-dimensional index of felt connectedness that may sensitively measure therapeutically relevant psychological changes post-psychedelic use. We believe that the operational definition of connectedness captured by the WCS may have broad relevance in mental health research.

**Supplementary Information:**

The online version contains supplementary material available at 10.1007/s00213-022-06187-5.

## Introduction

“Rarely, if ever, are any of us healed in isolation, healing is an act of communion”

— bell hooks

The psychological concept of felt connection or ‘connectedness’^1^ has been discussed previously in the context of psychedelic therapy (Thomas et al. 2013; Watts et al. 2017; Yaden et al. 2017), but has not yet been clearly defined. Participant accounts of psychedelic therapy often include reference to an increased sense of connectedness with one’s own senses, body and emotions; to friends, family and community; and to nature, the living world, global humanity, purpose and meaning. Here, we offer a definition of connectedness as ‘a state of feeling connected to self, others and the wider world’.

Many have argued that a sense of felt connection to self, other people and the world around us has a profound effect on our individual and collective wellbeing (Alexander 2008; Cacioppo and Cacioppo 2018; Hari 2019; Sorajjakool et al. 2008). Different types of connectedness have been defined and measured previously, e.g. social connectedness (Aron et al. 1992; Lee and Robbins 1998; Mashek et al. 2007), nature connectedness (Mayer and Frantz 2004; Nisbet et al. 2009) and a connection to spiritual values or transpersonal connection (Piedmont 2012; Reed 1991; Yaden et al. 2017, 2019). However, here, we hypothesise that each of these domains of connectedness may be linked by a common general factor that can simply be referred to as ‘connectedness’. We also propose that the different domains of connectedness may inter-relate and, potentially, interact, i.e. sharing a common relation with a perhaps latent, central or global connectedness factor.

Connectedness as a construct spanning these three domains has not been measured, or systematically studied. Yet the experience of feeling connected to self and others, and the interconnected living world, is commonly described by psychedelic ‘users’—whether they use be in ceremonial (Dobkin de Rios 1984; Gearin 2016; Kettner et al. 2021; La Barre 1938/1975; Tramacchi 2000), festival (Forstmann et al. 2020; St John 2009), dance party or ‘rave’ (Newson et al. 2021) or therapeutic contexts (Agin-Liebes et al. 2021; Argento et al. 2019; Belser et al. 2017; Loizaga-Velder and Verres 2014; Noorani et al. 2018; Swift et al. 2017; Thomas et al. 2013).

Previous research has highlighted the potential three-dimensional structure of a ‘connectedness’ construct. Specifically, in a study of ayahuasca-assisted therapy for addiction, quotes from interviews conducted with participants were grouped into the categories of ‘connection with self, connection with others, connection with spirit and nature’ (Argento et al. 2019); themes which corresponded very closey to findings of other ayahuasca research (Thomas et al. 2013; Trichter et al. 2009). Furthermore, previous psychedelic studies have found that aspects of *acute* psychedelic experience—which include connectedness (emotional, social or spiritual)—mediated longer term mental health benefits (Kettner et al. 2021; Roseman et al. 2019, 2017; Yaden and Griffiths 2020).

Just as psychedelic interventions may increase connectedness across the domains, so too may non-psychedelic interventions; a 2-week long ‘nature noticing’ intervention was found to increase not only connectedness to nature, but also to other people and life as a whole, based on survey responses (Passmore and Holder 2017). Another study found a correlation between nature connectedness and social connectedness scores in a sample of 327 people (Lee et al. 2015). Mindfulness and loving-kindness meditation exercises have been found to increase scores on social connectedness and nature connectedness measures, even though the interventions did not focus specifically on nature (Aspy and Proeve 2017). Other non-psychedelic studies using measures of personal connectedness, social connectedness or nature connectedness have found them to be associated with wellbeing and flourishing in healthy populations, and different types of connectedness have been identified as key predictors and mediators of psychological wellbeing (Capaldi et al. 2015; Cervinka et al. 2012; Lee et al. 2008; Ryff and Keyes 1995; Saeri et al. 2018; Seligman 2012; Zelenski and Nisbet 2014). As prosocial behaviour has been associated with serotonergic functioning (Crockett 2009), some proponents of pharmacological interventions might explore their use to increase connectedness.

The suggestion of the potential value of measuring connectedness as a construct comprising multiple co-occurring domains, rather than measuring these aspects separately, was inspired by qualitative analysis of post-treatment interviews with participants in a clinical trial of psilocybin therapy for treatment-resistant depression (Watts et al. 2017) where reports of a post-treatment shift from feeling disconnected to self, other people and the world—to feeling fundamentally more connected—were especially common and strongly emphasised. Many participants who experienced this feeling of connectedness after psilocybin described the treatment as working in a different way to antidepressants they had tried before, which were described by many as having actually exacerbated a sense of disconnection from self, others and the world (similar to emotional blunting (Price et al. 2009)). This observation implies a degree of orthogonality or independence between depressive symptoms severity and connectedness. After psilocybin, few people reported feeling more connected in a singular domain but rather described it as applying in a generalised way. This generalised connected state was reported as feeling most intense in the weeks to months after psilocybin therapy and occurred alongside a temporary reduction in depression scores (Carhart-Harris et al. 2017). Witnessing this association gave rise to the hypothesis that depression can be linked to a fundamental, multidimensional and generalised sense of disconnectedness and that psychedelic therapy (and other interventions) can bring about an increase in this generalised connectedness (Carhart-Harris et al. 2018; Watts et al. 2017).

We infer that connectedness holds transdiagnostic relevance both in relation to psychedelic therapy specifically (Kočárová et al. 2021) and beyond it (Carhart-Harris et al. 2018) in a similar way to other candidate transdiagnostic factors, such as psychological flexibility (Close et al. 2020; Davis et al. 2020a; Kashdan and Rottenberg 2010; Watts and Luoma 2020). Psychological flexibility (Hayes et al. 2011; Kashdan and Rottenberg 2010) and connectedness are proposed to overlap (Watts and Luoma 2020). Psychological flexibility includes accessing a ‘larger’ self rather than a smaller egoic self, and the ability to engage effectively with one’s senses and feelings in order to be guided towards value-led actions, rather than being trapped by habitual and unhelpful patterns of thinking. Thus, psychological flexibility typically focuses on the experience of the individual and relates more closely with ‘connectedness to self’. Connectedness includes ‘connectedness to self’, but more strongly emphasizes connectedness to others and world, incorporating inter-relatedness with community and environment into concepts of wellbeing.

Some questionnaires that measure the *acute* psychedelic experience include emotional, social and spiritual aspects of connectedness (Kettner et al. 2021; MacLean et al. 2012; Roseman et al. 2019; Yaden et al. 2019), but there are no measures to capture the multidimensional connectedness which may be felt in the weeks after a psychedelic experience and thus no tools to measure when this state begins to fade. The Watts Connectedness Scale (WCS) has been developed to enable measurement, with a single tool, of ‘connectedness to self, others and world’ in daily life, before and after any intervention which might be hypothesized to improve it, including psychedelic therapy. Whether or not the formulation of connectedness proposed by the WCS will have validity and usefulness is yet to be known; this measure has been created in order that we might have a tool to answer that question. If the separate aspects of connectedness previously studied are found to co-occur, then it will be important to define, measure and understand connectedness as a construct that is itself interconnected across its own domains.

In order to test the hypothesis that increased connectedness to self, others and world is a key mechanistic factor explaining therapeutic improvements post psychedelic therapy, we devised a new self-rated scale, the WCS, based on the findings of a thematic analysis of participant accounts of psychedelic therapy (Watts et al 2017) and applied it in three independent studies. This is the first scale that emerges from psychedelic research which is not measuring the acute experience, but the changes that happen after the experience. Validation of the WCS was done by means of conducting exploratory and confirmatory factor analysis on scores for pre-selected items, as well as reliability analysis. Data were pooled from two prospective online surveys completed pre- and post-psychedelic experiences (*N* = 1226). These data were used to assess the internal structure of the scale. We evaluated construct validity of the WCS by correlating WCS total and subscale scores with scores on related measures and tested postdictive criterion validity using measures of the spiritual, emotional and social components of the acute psychedelic experience. Specifically, the Mystical Experience Questionnaire (MEQ) (MacLean et al. 2012; Pahnke and Richards 1966), Emotional Breakthrough Inventory (EBI) (Roseman et al. 2019) and Communitas Scale (Kettner et al. 2021) were examined as predictors of changes in connectedness pre- vs post-psychedelic. We also evaluated sensitivity to change of the WCS in a randomised controlled trial that compared psilocybin-assisted therapy with the same therapy protocol delivered without the psilocybin, and with 6 weeks of daily escitalopram (an SSRI antidepressant medication). The therapeutic intervention that both groups received is outlined in detail in the ACE (Accept, Connect, Embody) manual (Watts 2021).

Specific hypotheses were as follows:

- H1: WCS will assess a principal, generalised connectedness factor with 3 subtypes or factors, namely, connectedness to self, connectedness to others and connectedness to the world (a second-order latent variable subsuming all three subscales will show high reliability, suggesting the three subscales can converge into one construct.)
- H2: Criterion validity—WCS scores will increase after psychedelic use.
- H3: Criterion validity (postdictive)—the acute experience (measured with MEQ, EBI and Communitas) will predict WCS increases.
- H4: Changes of WCS scores will be greater following psilocybin than escitalopram, measured in a controlled clinical trial.
- H5: Changes in WCS scores will be greater in psilocybin responders than escitalopram responders (response is defined by a baseline to primary endpoint reduction of at least 50% in depression symptoms measured on the primary outcome QIDS).

## Study 1: psychometric validation

### Methods

The data used in the psychometric validation procedure were obtained from two prospective, observational online surveys, the Global Psychedelic Survey (GPS) and the Ceremony Study (CS) (Kettner et al. 2021), investigating psychedelic use in real-world settings and in ceremony or retreat settings, more specifically in case of the latter. Both studies were approved by the Imperial College Research Ethics Committee and the Joint Research Compliance Office at Imperial College. Five other papers on different topics were published using data from these same studies (Kettner et al. 2021; Kuc et al. 2021; Spriggs et al. 2021; Timmermann et al. 2021; Zeifman et al. 2020).

### Participant recruitment

The survey studies collected data about self-selected individuals’ psychedelic experiences using an observational prospective cohort design. Individuals could participate in either study if they were at least 18 years old, had a good comprehension of the English language and if they intended to take a classic psychedelic within 2 months. Participants who planned to take a psychedelic via their own initiative (GPS) or within a planned ceremony or retreat setting (CS) could sign up online via the platform www.psychedelicsurvey.com. After giving informed consent, participants received email reminders including links to the surveys at different time points before and after the indicated date of the experience. The surveys were completed online on the platform SurveyGizmo. All obtained data were anonymous, and no personally identifying information was collected apart from e-mail addresses—which we required for participants to be sent the survey links. No data that could identify individuals or their responses have been shared, and no IP addresses were collected.

### Study design

Only measures relevant to the current analyses are reported here. In both studies, a baseline survey was completed 2 weeks prior to the scheduled experience. This included demographic information, psychological trait variables related to connectedness and the 23-item WCS (see below). In case of the ceremony study, a second time point was then completed by participants the day after the psychedelic session to assess, retrospectively, acute subjective experiences linked to the psychedelic. The WCS was repeated at three endpoints to assess sensitivity to change and postdictive validity: 2 weeks, 4 weeks, and again 6 months following psychedelic use. Data from a pooled sample of 1293 participants were analysed: 886 from the Ceremony Study and 407 from the Global Psychedelic Survey.

### Measures

#### Watts Connectedness Scale

Items of the Watts Connectedness Scale (WCS) were inspired by the results of a thematic analysis of 6-month follow-up interviews (Watts et al. 2017) done with twenty participants from a psilocybin therapy for treatment resistant depression open-label trial (Carhart-Harris et al. 2016). The first theme that emerged from the interviews was a change process of moving from a state of disconnection to connection (17/20). Participants described becoming more connected to senses (10/20), to themselves (16/20), to others (16/20), to the world (16/20) and to a spiritual principle (9/20). Below are few excerpts from the interviews which portray increased connectedness in the different domains:

*A veil dropped from my eyes, things were suddenly clear, glowing, bright. I looked at plants and felt their beauty. I can still look at my orchids and experience that: that is the one thing that has really lasted. (P3)*

*Things look different even now. I would look over at the park and it would be so green, a type of green I’d never experienced before. Being among the trees was incredible, like experiencing them for the first time, so vibrant, so alive. (P12)*

*[My wife and I] went for dinner for the first time in 6 years: we were like a couple of teenagers. (P16)*

*I would look at people on the street and think “how interesting we are”—I felt connected to them all. (P3)*

*Before I enjoyed nature, now I feel part of it. Before I was looking at it as a thing, like TV or a painting. You’re part of it, there’s no separation or distinction, you are it. (P1)*

Based on these qualitative findings, twenty-three items were chosen pertaining to connectedness, which we hypothesised would cluster into 3 dimensions, namely, connectedness to self, connectedness to others and connectedness to the wider world and spirituality. Five experts (academic and clinical professionals with psychology backgrounds, working in psychedelic research) assessed the items and revised them if needed (R Watts, L Roseman, M Kaelen, M Nour, and R Carhart-Harris). Items were rated on a 0–100 visual analogue scale (VAS), where 0 corresponded to ‘not at all’ and 100 to ‘entirely’. The instruction for the scale reads as follows: ‘Reflecting on how you have felt over the past 2 weeks, please rate the following items on a scale from “Not at all” to “Entirely” according to how you have felt over this time period. Please answer every item, even if you are unsure or feel the item is unclear or poorly worded. Drag the indicator to a position on the scale that shows how much you agree or disagree with each of the following statements’.

#### Previously validated measures of connectedness and wellbeing

##### *Social connectedness*

The Social Connectedness Scale (SCS) (Lee and Robbins 1998) consists of 8 items measuring belongingness, on a 6-point Likert scale.

##### *Nature relatedness*

The NR-6 (Nisbet and Zelenski 2013) is a short-form version of the Nature Relatedness Scale (NR) that measures connection with nature on a 5-point Likert scale.

##### *Experiential avoidance*

The Brief Experiential Avoidance Questionnaire (B-EAQ) (Gámez et al. 2014) contains 15 items that measure behavioural avoidance, distress aversion, procrastination, distraction/suppression, repression/denial and distress endurance, on a 6-point Likert scale.

##### *Flourishing*

Flourishing was measured using Flourishing Scale (Diener et al. 2010), which assesses perceived success and competence in areas spanning relationships, self-esteem, meaning and purpose in life through eight item rated on a 7-point Likert scale.

##### *Wellbeing*

The 14-item Warwick-Edinburgh Mental Wellbeing Scale (WEMWBS) (Tennant et al. 2007) was included to measure psychological wellbeing. Responses were rated on a 5-point Likert scale.

##### *Trait anxiety*

The Spielberger State-Trait Anxiety Inventory’s Short-Form Trait Version (STAI-SF) (Marteau and Bekker 1992) is a 6-item scale scored on a 4-point Likert scale.

#### Acute psychedelic experience

Few measures were used to assess the acute psychedelic experience. These were administered retrospectively, 1 day after the experience. The measures represent the acute experience of connection on the three dimensions and hence are potential mediators for long-term changes.

##### *Emotional breakthrough*

The Emotional Breakthrough Inventory (EBI) (Roseman et al. 2019) assesses moments of emotional breakthrough, catharsis or release. It is a 6-item scale scored on 0 to 100 visual analogue scale (VAS).

##### *Mystical‑type experience*

The Mystical Experience Questionnaire (MEQ) (MacLean et al. 2012) is a 30-item scale scored on a 5-point Likert scale. It consists of four factors which measure different dimensions of the mystical-type experience: ‘mystical’, ‘positive-mood’, ‘transcendence of time and space’ and ‘ineffability’.

##### *Communitas*

The Communitas Scale (COMS) (Kettner et al. 2021) assesses moments of being in harmony with the group and feeling a sense of belonging and connection to the group. It is an 8-item scale scored on a 7-point Likert scale.

### Statistical analysis

#### Psychometric validation of the WCS

Exploratory and confirmatory factor analyses (EFA and CFA, respectively) were performed to determine and test the factor structure of the WCS. The GPS sample (*N* = 407) was used for EFA, the larger CS sample (*N* = 819) for CFA.

#### Exploratory factor analysis (EFA)

As a first step, the number of factors to be extracted was identified through a combination of several heuristics, including visual examination of the scree plot, the ‘Kaiser rule’ (Kaiser 1960), which accepts as reliable factors, those whose corresponding eigenvalue is larger than one, optimal coordinate, parallel analysis and acceleration factor tests (Raîche et al. 2013). Kaiser–Meyer–Olkin (KMO) measure of sampling adequacy and Bartlett’s test of sphericity were used to test the sample adequacy for EFA. Maximum likelihood estimation was chosen to allow for continuity with CFA results. The selected rotation was oblimin, as this rotation method favours interpretability and allows factors to intercorrelate. The subscales were based on the factors extracted and interpreted through investigation of covariance-based factor loading patterns. To maximise clarity and simplicity in interpretation, two guiding criteria were considered. Each item assigned to a subscale should demonstrate a minimum factor loading of at least 0.3 (Field 2013) and maximum cross loading on any other factor should be 0.2 (Gaskin and Richard 2012).

#### Confirmatory factor analysis (CFA)

Based on the factor structure determined through EFA, three first-order latent variables were included, as well as an additional second order latent variable (WCS total) that was aimed to capture correlations between the first-order subscales, which were all set to load onto this second-order variable. To account for potential method effects, the error terms between negatively worded items were allowed to correlate. Metrics of each latent variable were determined by fixing the loading of the first item to 1.0 for each factor. Acceptable model fit was determined through a combination of fit indices, including the comparative fit index (CFI > 0.9), standardised root mean square residual (SRMR < 0.08) and root mean square error of approximation (RMSEA < 0.08). Coefficient Cronbach’s alpha was used to assess internal consistency of the latent variables, with > 0.8 and > 0.9 representing high and excellent reliability, respectively (Cronbach 1951). Additionally, composite reliability was calculated. It is less prone to over- or underestimations of reliability at a population level than Cronbach’s alpha, with a threshold of 0.7 suggesting acceptable consistency (Hair Jr et al. 2014).

#### Construct validity

The construct validity of the WCS was evaluated by inspecting how total and subscale WCS scores related to other previously validated measures. Pearson’s correlations were calculated between WCS components and other relevant measures taken at baseline within the ceremony study sample (*N* in analysis = 819), including the B-EAQ, SCS, NR-6, FS, WEMWBS and STAI-SF. Resulting correlations were interpreted based on effect sizes: negligible (up to − 0.1; 0.1), low (between − 0.1 and − 0.3; between 0.1 and 0.3), moderate (between − 0.5 and 0.3; between 0.3 and 0,5), and high (from − 0.5; 0.5) (Chen et al. 2010).

#### Criterion validity

In order to test whether the psychedelic experience influenced WCS scores in the Ceremony Study data, linear mixed models (LMM) were fitted to assess longitudinal changes in WCS and its subscales. The LMM used restricted maximum likelihood estimation and included in each case the WCS (subscale) score as dependent variable, time as a fixed factor and a random intercept to account for individual differences between participants. Eta squared effect sizes are reported for each model, where values of 0.02, 0.13 and 0.26 were considered to be small, medium and large, respectively (Cohen 1988). Postdictive validity was then assessed through Pearson correlations between changes on WCS total score between baseline and 2 weeks post-experience and measures of the acute psychedelic experience, including MEQ, EBI and COMS.

All statistical analyses were conducted in RStudio (v1.2).

### Results

#### Demographics

For demographics, see Table 1.

**Table 1:** Demographic information of both observational survey studies collected at baseline

		CS	GPS
Total *N*		819	407
Age		44.4 ± 12.6	30.46 ± 10.5
Gender	Female	359 (43.8%)	123 (30.2%)
	Male	455 (55.6%)	277 (68.1%)
	Other	5 (0.6%)	7 (1.7%)
Nationality	United States	359 (43.8%)	95 (23.3%)
	United Kingdom	160 (19.5%)	107 (26.3%)
	Australia	31 (3.8%)	8 (2.0%)
	Germany	28 (3.4%)	21 (5.2%)
	Canada	26 (3.2%)	31 (7.6%)
	Other countries	215 (26.3%)	145 (35.6%)
Education	None	6 (0.7%)	38
	High school or equivalent (GED)	62 (7.6%)	160 (39.3%)
	Associate/technical Degree	58 (7.1%)	N/A*
	College diploma	250 (30.1%)	118 (29.0%)
	Master’s degree	275 (33.6%)	91 (22.4%)*
	Doctorate or Professional degree	168 (20.5%)	
Employment	Student	46 (5.6%)	129 (31.7%)
	Working full-time	520 (63.4%)	186 (45.7%)
	Working part-time	120 (14.7%)	49 (12.0%)
	Retired	73 (8.9%)	7 (1.7%)
	Unemployed	60 (7.3%)	36 (8.8%)
Median household income ($/month)		9000	3200
Ethnicity	White	743 (90.7%)	372 (91.4%)
	Black or African American	12 (1.5%)	5 (1.2%)
	Asian	48 (5.9%)	22 (5.4%)
	American Indian or Alaska native	3 (0.4%)	6 (1.5%)
	Unknown/prefer not to say	11 (1.3%) / 23 (2.8%)	10 (2.5%) / 16 (3.9%)
Marital status	Cohabiting with partner	101 (12.3%)	70 (17.2%)
	Married	340 (41.5%)	60 (14.7%)
	Divorced	86 (10.5%)	18 (4.4%)
	Separated	29 (3.5%)	10 (2.5%)
	Never married	254 (31.0%)	247 (60.7%)
	Widowed	9 (1.1%)	1 (0.2%)
Previous psychedelic use	Never	330 (40.3%)	48 (11.8%)
	Once	95 (11.2%)	26 (6.4%)
	2–5 times	166 (20.3%)	91 (22.4%)
	6–10 times	73 (8.9%)	78 (19.2%)
	11–20 times	76 (9.3%)	65 (16.0%)
	21–50 times	49 (6.0%)	54 (13.3%)
	> 50 times	30 (3.7%)	45 (11.1%)

Absolute frequencies including corresponding percentages (in brackets) are presented

Plus–minus values are means ± SD

*CS* Ceremony Survey; *GPS* Global Psychedelic Survey

For more information on CS dataset, see Kettner et al. (2021)

^*^In the GPS sample, ‘associate/technical degree’ was not assessed separately; ‘postgraduate, master’s and doctorate degree’ were combined into one response option

#### Factor structure

All 23 original WCS items were entered into a maximum likelihood–based exploratory factor analysis (EFA). The global psychedelic survey (GPS) dataset was found to be suitable for factor analysis, as its Kaiser-Meyer-Olkin (KMO) measure of sampling adequacy was 0.92 and Bartlett’s test of sphericity was significant (χ2(22) =188.75, *p*<0.0001) (Budaev 2010). Although a fourth eigenvalue was found to be above 1 (at 1.007), visual examination of the scree plot (see supplementary Figure S1), parallel analysis and optimal coordinate estimates of the ideal number of factors to be extracted pointed to a 3-factorial solution, which was chosen for the subsequent extraction of factor loadings. Item loading patterns (supplementary table S1) revealed that four items had significant cross loading (> 0.2), which were therefore removed and the EFA repeated. These excluded items were’“I have felt connected to deeper aspects of myself’, ‘I have felt connected to insight intuition’, ‘I have felt connected to my values’, and ‘I have felt connected to strangers’. The remaining 19 items all had satisfactory loading patterns, where the first factor explained 22% of the variance, the second factor 16% and the third factor 12%, amounting to a total of 50% of variance explained in the final 3-factorial solution. Based on the content of items that loaded on each factor, definitions were assigned that each represented a subtype of connectedness, in line with the hypothesised 3-factorial structure of connectedness (supplementary table S1). Accordingly, the first factor was named ‘connectedness to world’ (CTW), the second factor was named ‘connectedness to self’ (CTS), and the third factor was named ‘connectedness to others’ (CTO)

**Fig. 1:**
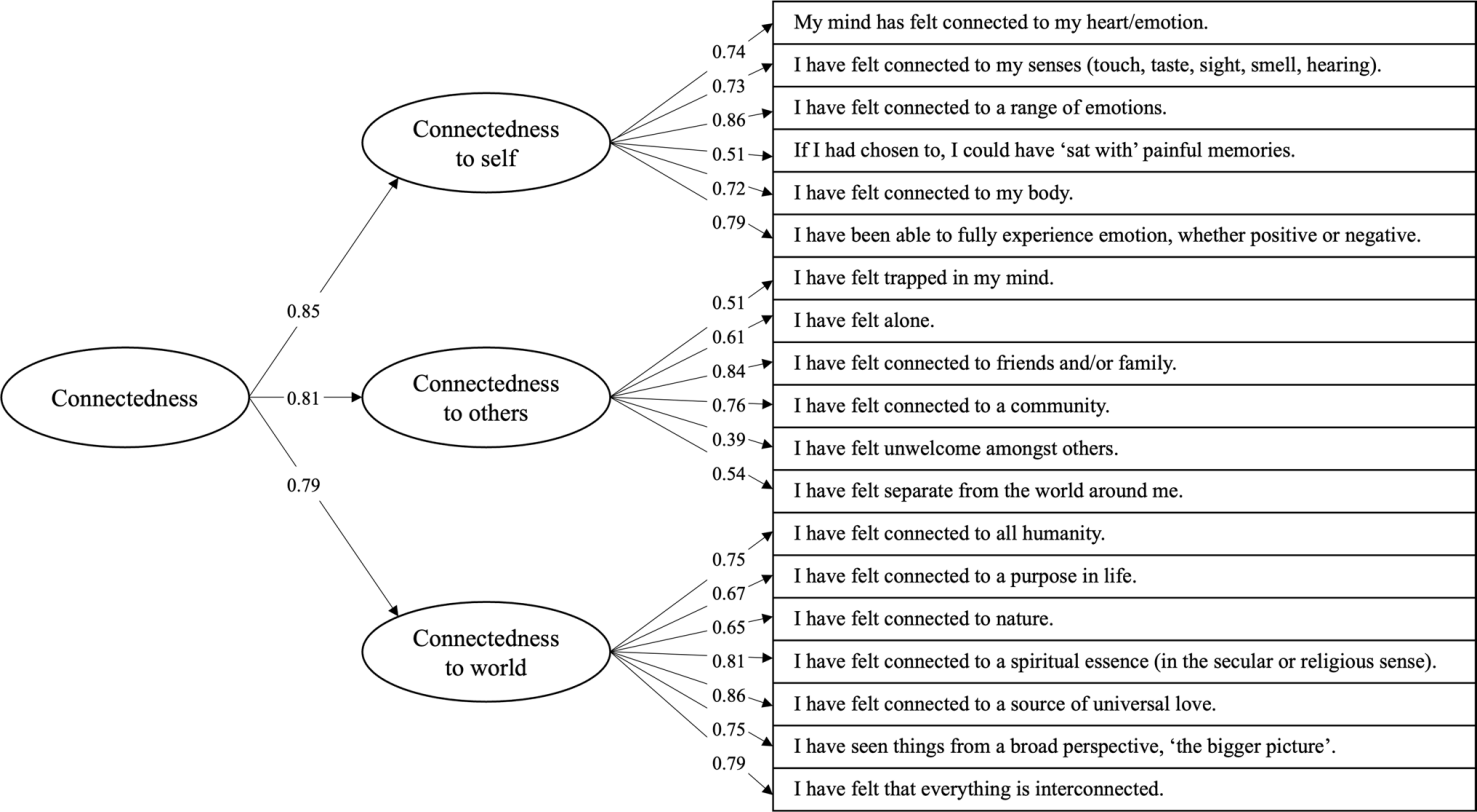
Confirmatory factor analysis model with standardised loadings. Included in the model (*N* = 819) were 19 items of the Watts Connectedness Scale which fulfilled loading criteria during exploratory factor analyses in a different sample (*N* = 407). Error terms between negatively worded items were allowed to correlate to account for method effects

The 3-factorial structure consisting of 19 remaining items showed satisfactory psychometric properties in the EFA and then subjected to a subsequent confirmatory factor analysis (CFA) in the separate Ceremony Study dataset (*N*=819), to which a second-order WCS total score was added onto which the three latent subscales were set to load. The model showed acceptable fit with CFI = 0.902, RMSEA = 0.076 (confidence intervals 0.072–0.081) and SRMR = 0.060 (Maiyaki 2012). Standardised factor loadings of the resulting model are shown in Figure 1. Cronbach’s alphas for the three first-order latent factors were 0.84, 0.87 and 0.90, while composite reliabilities were 0.79, 0.87 and 0.90, for connectedness to self, others and the world, respectively. The second-order latent variable subsuming all three subscales had a composite reliability of 0.86.

From here on, the total score of each of the subscales was calculated by averaging all of the items that belong to this subscale, and the total WCS score was calculated by averaging the three subscales.

#### Construct validity

Correlation coefficients between the WCS, its subscales and other included measures are displayed in Table 2.

**Table 2:** Pearson-correlation coefficients between the Watts Connectedness Scale, its subscales and validated secondary measures

	WCS_Total_	CTS	CTO	CTW	bEAQ	FS	SCS	NR-6	STAI	WEMWBS	$\sqrt{AVE}$
WCS_Total_	1.00	0.85	0.80	0.86	− 0.57	0.71	0.69	0.36	− 0.61	0.79	
CTS	0.88	1.00	0.52	0.62	− 0.53	0.51	0.49	0.28	− 0.46	0.58	0.72
CTO	0.67	0.52	1.00	0.51	− 0.51	0.72	0.78	0.15	− 0.67	0.82	0.62
CTW	0.90	0.62	0.51	1.00	− 0.39	0.55	0.47	0.46	− 0.42	0.60	0.76

*WCS* Watts Connectedness Scale; *CTS* connectedness to self; *CTO* connectedness to others; *CTW* connectedness to the world; *bEAQ* Brief Experiential Avoidance Questionnaire; *FS* Flourishing Scale; *SCS* Social Connectedness Scale; *NR-6* Nature Relatedness Scale; *STAI*: State-Trait Anxiety Inventory; *WEMWBS* Warwick-Edinburgh Mental Wellbeing Scale; *AVE* average variance extracted

Discriminant validity could be established among the WCS subscales following the Fornell and Larcker (1981) criterion; i.e. the square root of the average variance extracted (AVE) was larger than any inter-construct correlations, although correlations among WCS subscales were generally high, ranging from 0.51 (CTO and CTW) to 0.62 (CTS and CTW). The highest correlations with secondary measures were observed between CTO and wellbeing (WEMWBS, *r* = 0.82), social connectedness (SCS, *r* = 0.78) and flourishing (FS, *r* = 0.72), which were greater than the square root of the AVE, meaning that discriminant validity for this subscale could not be established against the mentioned secondary measures.

#### Criterion validity

**Fig. 2:**
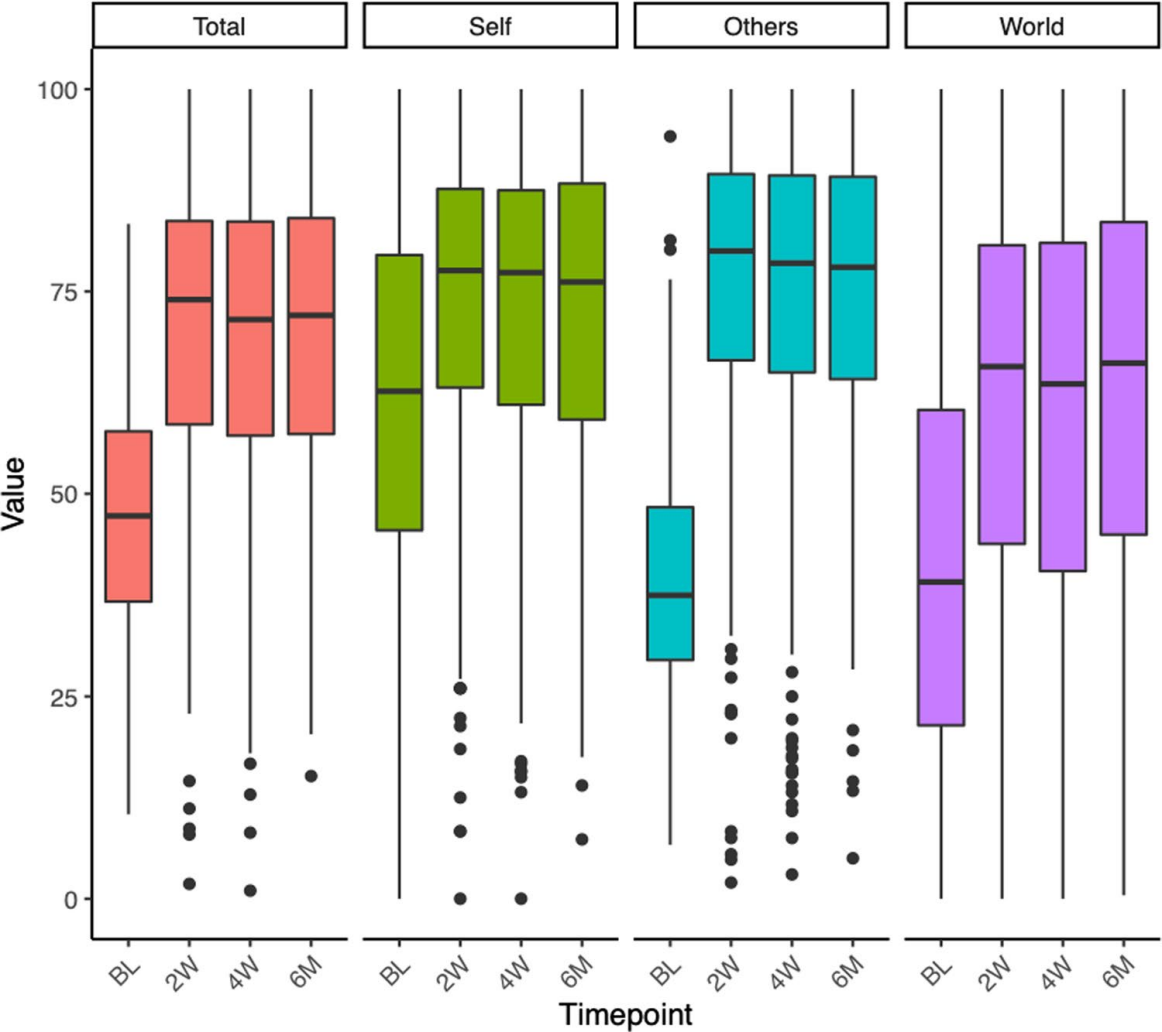
Changes in connectedness following a psychedelic experience in a guided group setting. Connectedness across all subscales was significantly (*p* < .0001) enhanced 2 weeks, 4 weeks and 6 months following the experience compared to baseline. Error bars indicate 95% confidence intervals. CTO, connectedness to others; CTS, connectedness to self; CTW, connectedness to world; WCS, Watts’ Connectedness Scale (total)

**Fig. 3:**
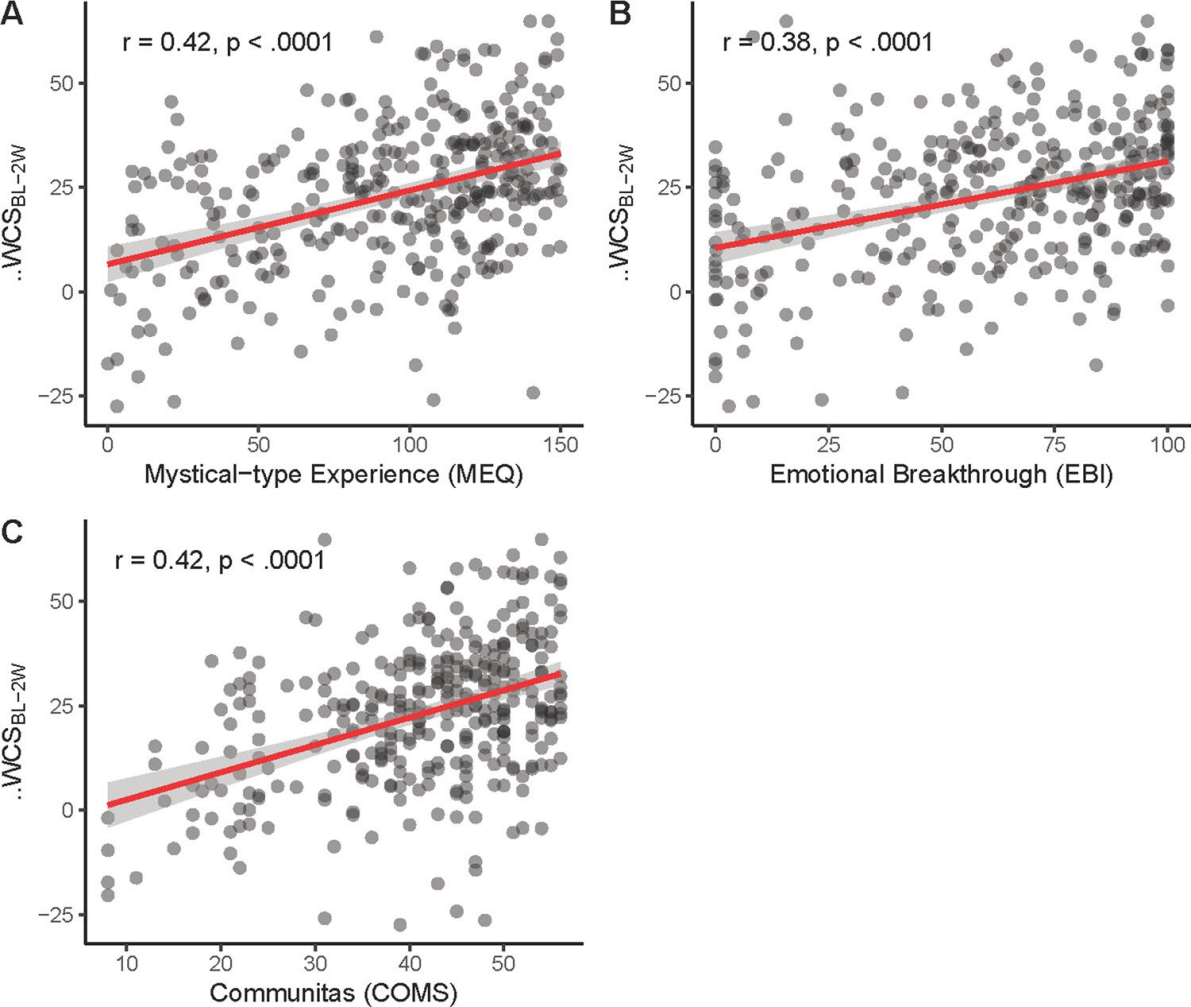
Correlations between **A** mystical-type experiences, **B** emotional breakthrough, and **C** communitas and change scores on Watts Connectedness Scale (WCS) from before to after 2 weeks of a guided psychedelic group experience

Mixed linear models were fitted to test for changes in connectedness across time in the Ceremony Study data. Parameter estimates for each time point and subscale are presented in Table 3; all values were significantly (*p* < 0.0001) increased at 2 weeks, 4 weeks and still at 6 months following the psychedelic experience (Fig. 2), with consistently large effect sizes ranging from *η*^2^ = 0.192 for connectedness to self to *η*^2^ = 0.339 for changes in the overall WCS score. Estimation of individual contrasts showed that for each subscale, 2 weeks, 4 weeks and 6 months time points differed from baseline at *p* < 0.0001; additionally, the overall WCS score was significantly lower at 6 months, compared with 2 weeks (mean difference = 3.28, se = 1.25, *p* = 0.027), as was connectedness to others (mean difference = 4.72, se = 1.41, *p* < 0.01). Postdictive validity was good; and Pearson correlations between changes in WCS total scores from baseline to 2 weeks and measures of the acute psychedelic experience were significant in the case of mystical-type experience (MEQ, *r* = 0.42, *p* < 0.0001), emotional breakthrough (EBI, *r* = 0.38, *p* < 0.0001) and communitas (COMS, *r* = 0.42, *p* < 0.0001) measured retrospectively 1 day following the psychedelic ceremony (Fig. 3).

**Table 3:** Mixed linear effect model results of WCS subscales across time

	WCS_Total_	CTS	CTO	CTW
Intercept	54.31 (0.67)***	61.34 (0.73)***	59.88 (0.75)***	41.69 (0.88)***
*t* = 2 weeks	16.21 (0.78)***	12.84 (0.89)***	15.52 (0.88)***	20.26 (1.09)***
*t* = 4 weeks	14.84 (0.79)***	11.66 (0.90)***	13.68 (0.89)***	19.19 (1.05)***
*t* = 6 months	12.93 (1.2)***	9.77 (1.38)***	10.8 (1.36)***	18.32 (1.6)***
Effect size *η*^2^	0.339	0.192	0.260	0.332

*WCS* Watts Connectedness Scale; *CTS* connectedness to self; *CTO* connectedness to others; *CTW* connectedness to world

***It means *p* < 0.0001

### Discussion Study 1

The observed factor structure confirmed the three-factorial structure of connectedness, including a connection to self, connection to others and connection to the world. The dimensions of the scale were in line with the hypothesised constructs. The factor loadings showed a simple structure with minimal cross loadings, which promoted a straightforward interpretation of scores (Worthington and Whittaker 2006), and the internal consistency of the scale was high. Most importantly, in the confirmatory factor analysis, the second-order total WCS score, which is comprised of the three subscales, had a high composite reliability. This result suggests that the three subscales can be converged into one construct which measures a comprehensive spectrum of connectedness.

The construct validity of the scale was assessed via comparison with other related scales that measured psychological flexibility, wellbeing, social connectedness, nature relatedness and anxiety. High correlations were observed between total WCS and its subscales and scores on the other measures, showing high convergent validity with these scales. The high convergent validity with a variety of scales implies a broad, multidimensionality to the WCS. Regarding divergent validity, CTS and CTW showed divergence from other measures, while CTO did not diverge from few of the other measures such as SCS and WEMWBS. Overall, we can conclude that the total score of WCS does diverge from other measures, yet there is still a need to investigate divergent validity by comparing WCS with other measures that were not included in the current study (e.g., the relation between CTW and different measures of spirituality).

As CTO did not diverge from SCS, it is important to note the relationship between them. The two scales converge in terms of their shared reference to the negative affect that can accompany social disconnection and only slightly differ on the degree to which actual social relationships exist. One minor difference is that SCS features items that assess social connectedness that go beyond one’s direct social environment. Total WCS also had high correlations with SCS, which most likely implies that social connectedness is an important aspect of connectedness. This finding follows the principal idea that humans are a species whose survival, thriving and meaning-making depend on good social relations (Hagerty et al. 1993; Townsend and McWhirter 2005). A recent big data study supported this notion by showing that social connection is the most crucial protective factor in preventing depression (Choi et al. 2020).

The postdictive validity of WCS was excellent. Previous psychedelic research showed that different psychedelic experiences can predict long-term changes, in naturalistic studies (Haijen et al. 2018; Kettner et al. 2021) and clinical studies (Griffiths et al. 2016; Roseman et al. 2017). Measures that were used in previous psychedelic research (MEQ, EBI and COMS) predicted in the current study long-term changes in WCS. This means that the emotional, social and spiritual components of the acute psychedelic experience impact the sense of connectedness for a prolonged period after the psychedelic has been metabolised. This result adds to a large body of findings showing that acute subjective effects induced by psychedelics are fundamental to the therapeutic efficacy induced by psychedelics (MacLean et al. 2011; Roseman et al. 2017; Schenberg 2018; Yaden and Griffiths 2020).

## Study 2: sensitivity and specificity of outcome in a psilocybin vs escitalopram RCT

### Methods

To test the WCS’ sensitivity to change and specificity of effect to psychedelic compounds, data from a double-blind randomised controlled trial (Carhart-Harris et al. 2021) was analysed in study 2. A Schedule 1 drug license from the UK Home Office was obtained by the investigators, and the trial was approved by the Brent Research Ethics Committee, the UK Medicines and Healthcare Products Regulatory Agency, the Health Research Authority, the Imperial College London Joint Research Compliance and General Data Protection Regulation Offices, and the risk assessment and trial management review board at the trial site (the National Institute for Health Research [NIHR] Imperial Clinical Research Facility [CRF]). Psilocybin (as COMP360) was provided by COMPASS Pathways, and escitalopram and placebo were provided by the Pharmacy Manufacturing Unit at Guy’s and St. Thomas’s Hospital.

### Participant recruitment

The inclusion criterion for the RCT was major depressive disorder (MDD) of a moderate to severe degree, diagnosed by a doctor, and with the participant scoring 17 + on the 21-item Hamilton Depression Rating scale [HAM-D])—as assessed by a study psychiatrist at the point of telephone screening. Exclusion criteria were current or previously diagnosed psychotic disorder; immediate family member with a diagnosed psychotic disorder; medically significant condition rendering unsuitability for the study; history of serious suicide attempts (requiring hospitalisation); history of mania; blood or needle phobia; positive pregnancy test at screening or during the study; and current drug or alcohol dependence; and any diagnosed or suspected psychiatric comorbidities felt to jeopardise the formation of a good therapeutic relationship. Information about the study’s recruitment was sent to general practitioners via the North West London Clinical Research Network. However, patients were also allowed to self-refer to the study if they were UK residents. Patients who initiated contact with the research team (via email, letter or telephone) were sent a study information sheet, a subsequent telephone screening and further face-to-face medical and psychological screening including liaison with all participants’ healthcare providers.

### Study design

This was a phase 2 double-blind randomised controlled trial. Participants were randomly assigned 1:1 to either a psilocybin condition or an escitalopram condition. Participants in both groups received the same therapeutic intervention which focused on enabling participants to accept and allow any difficult feelings, connect to any insights and engage with their embodied ‘felt sense’ throughout the therapy process. This approach is described in detail in the ACE (Accept, Connect, Embody) model manual (Watts 2021). The primary outcome measure was the change in self-rated scores on the Quick Inventory of Depressive Symptomatology (QIDS-16) from baseline to the primary endpoint. The primary endpoint was 6 weeks after the first psilocybin session and 3 weeks after the second psilocybin session (also the day of the final dose of 6 weeks of daily escitalopram). Secondary measures included the Hamilton Depression Rating Scale (HAM-D) and Montgomery-Asberg Depression Rating Scale (MADRS) clinical interviews to assess depression symptoms, the Beck Depression Inventory (BDI) self-rated measure of depressive symptom severity and other self-rated scales including the WCS. Secondary measures were administered at baseline and 6-week endpoint.

The psilocybin condition (*n* = 30) comprised two 4–6-h sessions in which participants consumed 25-mg psilocybin whilst listening to a therapeutic music playlist, under the guidance of two therapists who were allocated to them for the entirety of the trial. These sessions were 3 weeks apart. Participants in the psilocybin condition also took daily capsules, containing placebo, from the day after the first guided dosing session until the 6-week endpoint. The escitalopram condition (*n* = 29) comprised two 4–6-h sessions in which participants consumed a very small dose of psilocybin (1 mg) whilst listening to the same therapeutic music playlist, under the guidance of their two allocated therapists. These sessions were also 3 weeks apart. Participants in the escitalopram condition took daily capsules, containing the antidepressant medication escitalopram, from the day after the first guided dosing session until the 6-week endpoint. Participants in both groups received nine non-drug therapeutic sessions with their two allocated therapists which took place from 2 weeks before the first psilocybin session until the 6-week endpoint. A minority of the non-drug therapeutic sessions (which were termed ‘preparation’ and ‘integration’ sessions) took place with just one therapist via tele-health, but most included both therapists and took place face-to-face in a psilocybin therapy room. Both therapists were present during the entirety of both guided dosing sessions, for both conditions. The final non-drug therapeutic session at the 6-week endpoint was followed by a research interview in which participants completed primary and secondary outcome measures. It is important to note that participants in the escitalopram condition received the nine non-drug therapeutic sessions under the guidance of one or both of their two allocated therapist ‘guides’, and two 4–6-h guided psilocybin sessions with both therapist guides. Therefore, participants in both conditions received a large amount of psychotherapeutic support and personal attention.

### Measures

#### Depression

The Quick Inventory of Depressive Symptomatology (QIDS) was the primary outcome of the RCT (Carhart-Harris et al. 2021), and is used in the current paper to define responders and non-responders to both psilocybin and escitalopram. Response was defined as more than 50% reduction in QIDS.

#### Connectedness

In the original 23-item version of the WCS was included in the trial, but for the purpose of this validation paper, we used only the 19 items that load well onto the aforementioned 3 dimensions of connectedness i.e. the subscales CTS, CTO and CTW.

### Statistical analysis

Groups were split into responders and non-responders based on more than 50% reduction in baseline depression scores. Mixed repeated measures ANOVA (2 × 2 × 2 between-within) was used to measure whether WCS differed between psilocybin and escitalopram (hypothesis H4), and whether this was dependent on response rates (hypothesis H5). A significant triple interaction would suggest that psilocybin and escitalopram differ in their therapeutic mechanism and that this difference is related to connectedness. WCS scores were included as dependent variable, time as within-subject effect with 2 levels (baseline and 6 weeks) and condition and response as between-subject independent variables with 2 levels each (psilocybin vs. escitalopram; and responders vs. non-responders). The resulting three-way interaction was time X condition X response. Multiple pairwise comparisons were calculated to test simple main effects: within-group comparisons (from baseline to 6 weeks) at each level of the group factor (psilocybin vs. escitalopram) and between-group comparisons at each time-point (baseline and week 6) were calculated. To test that connectedness is part of the mechanism of action in psilocybin but not escitalopram, it was possible to use moderated mediation analysis. However, we decided not to do so, as WCS and QIDS were measured in the same time point (6 weeks), and accurate mediation analysis require that the mediating variable is tested before the outcome.

All statistical analyses were conducted in RStudio (v1.2).

### Results

#### Demographics

See Table 4.

**Table 4:** Demographic information of for psilocybin and escitalopram (after exclusion)

		Psilocybin	Escitalopram
Total N		27	25
Age		42.3 ± 11.9	39 ± 10.2
Gender	Female	10 (37%)	7 (28%)
	Male	17 (63%)	18 (72%)
White Ethnicity		25 (92.6%)	20 (80%)
Education	University level	20 (74.1%)	19 (76%)
Employment	Employed	18 (66.7%)	19 (76%)
	Student	2 (7.4%)	1 (4%)
	Unemployed	7 (25.9%)	5 (20%)
Duration of illness (years)		21.4 ± 10.8	15.3 ± 11.5
No. of psychiatric medications previously used		2.15 ± 1.63	1.96 ± 1.51
Previous use of psilocybin		2 (7.4%)	0 (0%)
Previous use of psychotherapy		25 (92.6%)	22 (88%)

Absolute frequencies including corresponding percentages (in brackets) are presented

Plus–minus values are means ± SD

For more information on this data set, see Carhart-Harris et al. (2021)

#### Changes in Connectedness

**Fig. 4:**
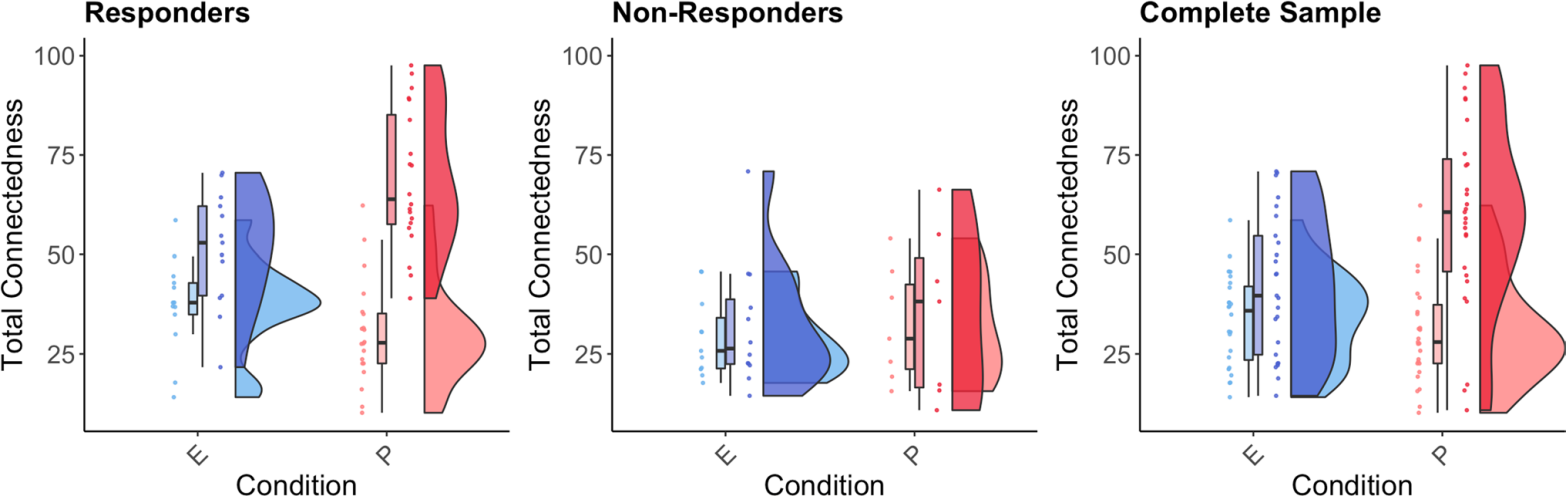
Change in connectedness in a randomised controlled trial comparing escitalopram (E) and psilocybin (P) for major depression from baseline to 6 weeks (endpoint). For the complete sample, significantly greater increases in connectedness were observed. Furthermore, a significant three-way interaction indicated significantly greater increases in connectedness for psilocybin-responders, compared to escitalopram responders, whereas non-responders did not increase in connectedness in either condition. Escitalopram arm = blue, psilocybin arm = red. Baseline = light colour, endpoint = dark colour

Four subjects in the escitalopram group came off the medication before the end of the trial and were excluded from the analysis. From the psilocybin group, one subject was excluded due to smoking cannabis on a regular basis during the trial period, and two were excluded for not having the second psilocybin dosing day due to COVID-19 lockdown restrictions. After exclusion, the escitalopram group consisted of 25 subjects and the psilocybin group, 27 subjects. Based on changes in QIDS scores, 13 subjects were defined as responders in the escitalopram group (52% of the total escitalopram group), and 20 in the psilocybin group (74% of the total psilocybin group). The mixed (2 × 2 × 2 between-within) rm ANOVA revealed a significant main effect of time (baseline vs 6 weeks) [*F*(1, 48) = 51.36, *p* < 0.0001, *η*_*p*_*2* = 0.517]; significant interaction effect for time and condition (escitalopram vs. psilocybin), aligned with hypothesis H4 [*F*(1, 48) = 7.39, *p* = 0.009, *η*_*p*_*2* = 0.133] (see Fig. 4); significant interaction effect for time and response (non-responders vs. responders) [*F*(1, 48) = 28.73, *p* < 0.001, *η*_*p*_*2* = 0.374]; and significant triple interaction for time, condition and response, aligned with hypothesis H5 [*F*(1, 48) = 9.62, *p* = 0.003, *η*_*p*_*2* = 0.167]. Two-sample *t*-tests were used to test whether WCS scores were different at baseline and at 6 weeks. No significant differences were observed at baseline for condition (mean difference = 1.8, Hedge’s *g* = 0.14, *p* = 0.614) and response (mean difference = 3.73, Hedge’s *g* = 0.29, *p* = 0.311), suggesting that the interaction effects were not driven by difference at baseline. Significant differences in ΔWCS scores were observed for condition (mean difference = 19.8, Hedge’s *g* = 1.07, *p* < 0.001, higher for psilocybin) and response (mean difference = 24.9, Hedge’s *g* = 1.45, *p* < 0.001, higher for responders). Paired samples *t*-tests were used to compare WCS at baseline vs. 6-week follow-up. There were no significant difference for both escitalopram non-responders (mean difference = 4.6, Hedge’s *g* = 0.42, *p* = 0.158, *n* = 12) and psilocybin non-responders (mean difference = 2.99, Hedge’s *g* = 2.5, *p* = 0.512, *n* = 7). There were significant differences for both escitalopram responders (mean difference = 14.3, Hedge’s *g* = 0.95, *p* = 0.004, *n* = 13) and psilocybin responders (mean difference = 38.58, Hedge’s *g* = 2.29, *p* < 0.001, *n* = 20). To test whether the change in WCS scores for psilocybin responders was higher than for escitalopram responders, as hypothesised, a two-sample *t*-test was used to compare the change in WCS scores. Aligned with hypothesis H5, ΔWCS was significantly greater for psilocybin responders compared with escitalopram responders (mean difference = 24.5, Hedge’s *g* = 1.52, *p* < 0.001), but not greater for psilocybin non-responders compared to escitalopram non-responders (mean difference = 1.6, Hedge’s *g* = 0.14, *p* = 0.76). Overall, results were consistent with our prior hypotheses, showing that changes in WCS were larger for psilocybin vs escitalopram and larger for psilocybin responders vs escitalopram responders (see Fig. 4). The significant triple interaction suggests that while both psilocybin and escitalopram can alleviate depression, psilocybin leads to stronger increases in connectedness even when comparing clinical responders from both groups. This suggests that the mechanism of action by which psilocybin and escitalopram improve depression is different and that this difference is related to connectedness which is higher for psilocybin.

Further descriptive analysis of the three dimensions was conducted. For escitalopram non-responders, CTS and CTO did not show significant changes (CTS, Hedge’s *g* =  − 0.064, *p* = 0.823; CTO, Hedge’s *g* = 0.37, *p* = 0.21), while CTW did show significant increase (Hedge’s *g* = 0.73, *p* = 0.023). For escitalopram responders, CTS did not show significant changes (Hedge’s *g* = 0.27, *p* = 0.34), while CTO and CTW did show significant increases (CTO, Hedge’s *g* = 1.46, *p* < 0.001; CTW, Hedge’s *g* = 0.79, *p* = 0.012). For psilocybin non-responders, none of the dimensions showed significant changes (CTS, Hedge’s *g* =  − 0.237, *p* = 0.476; CTO, Hedge’s *g* = 0.11, *p* = 0.739; CTW, Hedge’s *g* = 0.56, *p* = 0.113). For psilocybin responders, all of the dimensions showed significant increases (CTS, Hedge’s *g* = 1.85, *p* < 0.0001; CTO, Hedge’s *g* = 1.45, *p* < 0.0001; CTW, Hedge’s *g* = 1.4, *p* < 0.0001).

## Discussion

The result of our analyses support the validation of the WCS as an instrument to measure connectedness as a construct comprising three categories (connectedness to self, others, world). Aligned with our primary hypothesis, the validation of the scale has shown that those three categories can be grouped together into one single factor to measure a generalised type of connectedness. The WCS, and all of its subscales, showed significant increase up to 6 months after a psychedelic experience in three independent studies. Importantly, three different elements of the acute psychedelic experience—emotional, spiritual and social—predicted the long-term changes in WCS. Finally, in a double-blind randomised controlled trial comparing psilocybin-assisted therapy with escitalopram-assisted therapy for depression, the WCS showed robust post-treatment changes with psilocybin-assisted therapy that were significantly larger than for escitalopram-assisted therapy, even when comparing just the responders from both groups. These results confirm the hypothesis that was developed in the exploratory qualitative research which kindled the current quantitative investigation (Watts et al. 2017), which proposed that the therapeutic mechanism of psychedelic-assisted therapy is different to conventional antidepressants, with an increased sense of connectedness to self, others and the world being specific to psychedelic therapy.

### Connectedness as a construct

Many constructs which were originally conceptualised to be related to one particular aspect of connectedness (i.e. experiential avoidance to CTS, social connectedness to CTO, nature connectedness to CTW) were found to correlate with the total score of WCS. This supports the suggestion of a more general connectedness at the root of the various specific types of connectedness that have been previously studied. Based on the findings of this study, we now predict, for example, that an individual reporting feeling connected to a sense of meaning and purpose as well as their body and emotions would also report feeling connected to other people; or that someone describing feeling connected to nature would also report feeling connected to humanity at large and their own emotions. There will of course be exceptions to this, with a whole range of unique profiles of connectedness, but overall high scores on one domain (self/others/world) suggest high scores on others, just as low scores in one aspect suggest a disconnection across multiple domains. Extending on this theme, we hypothesise that high multidimensional connectedness may be a protective factor for mental health and wellbeing.

The construct of ‘connectedness’ has developed over time to have different meanings and different applications (Townsend and McWhirter 2005). Many older conceptualisations described connectedness as some form of interpersonal relatedness (Townsend and McWhirter 2005), some emphasised the self-in-relation-to-others context-dependent nature of connectedness (Hawkley et al. 2005; McWhirter 1990; Newcomb 1990; Newcomb and Bentler 1988), whereas others have favoured more internally focused experiential and emotion-oriented interpretations (Hagerty et al. 1993; Lee and Robbins 1995; Townsend and McWhirter 2005). Other types of connectedness have also been discussed in older literature, but have remained relatively underexplored. Bellingham et al. (1989) is a notable exception, conceptualising connectedness with three subtypes of connectedness (self, others and a larger purpose in life) which maps closely onto the model presented here. It is our hope that the WCS is able to capture both the core essence and multidimensional nature of the phenomenon of connectedness, and we hope that future use of this scale will help to revive an interest in the science of connectedness and its relevance to health.

### Connectedness to self

Psychedelic therapy tends to be experienced in a more embodied way than traditional talking therapy. During a 5–6-h psilocybin session, participants are encouraged to be with their felt sense in the body and refrain from speaking very much until afterwards, in order to more fully connect with somatic and emotional aspects of experience, rather than getting distracted by cognition or communication (Richards 2015). Participants engaging in psychedelic therapy tend to describe feeling connected to deeper aspects of themselves than they usually feel, and being able to ‘sit with’ more intense and often uncomfortable emotions than they can usually access or tolerate. Therefore, ‘self-connectedness’ in psychedelic therapy tends to be described as connectedness to the senses, the body, and emotions.

Previous conceptualisations of ‘self-connection’ have been largely cognitive, emotional and behavioural (Bellingham et al. 1989; Klussman et al. 2020, 2021) and have not included embodied/somatic aspects. CTS, as formulated by the WCS, acknowledges embodied qualities of ‘the self’, e.g., with the inclusion of the specific items ‘I have felt connected to my body’ and ‘I have felt connected to my senses’.

The original 23-item version of the WCS, CTS contained a number of items that map on to Klussman et al.’ s conceptualisation of CTS (values, insight, self compassion) but were removed because they were found to cross load with CTW: ‘I have felt connected to deeper aspects of myself’,‘I have felt connected to insight/intuition’ and ‘I have felt connected to my values’. A further item,‘I have felt connected to a purpose in life’, was hypothesised to load into the CTS subscale until factor analysis placed it in CTW. By removing these four items from the subscale, CTS becomes a more visceral, emotional and embodied connection to self. Of all CTS items in the original scale, the items with the highest factor loadings for CTS were those relating to emotions (i.e. the highest loading was for “I have felt connected to a range of emotions). This could be viewed as reflective of a differential ‘willingness to feel’ in the CTS subscale, as well as the core affective and interoceptive quality of CTS, as defined by this subscale’s six items.

### Connectedness to others

CTO maps closely on to the well-researched concept of social connectedness, which has been described by Lee and Robbins (1995) as being related to one’s view of the self in relation to others or as a 'cognitive structure representing regularities in patterns of interpersonal relatedness’ (Baldwin 1992 p. 461). One model of social connectedness is derived from a factor analysis of the UCLA Loneliness Scale (UCLA LS-R). Loneliness has been defined as the inverse of human (social) connectedness (Hawkley et al. 2005; Newcomb 1990; Van Bel et al. 2009). The UCLA LS-R has a three factor model: isolation, relational connectedness and collective connectedness (Hawkley et al. 2005). Relational connectedness refers to actual social networks, whereas isolation refers to an individual’s mental representations of how socially connected or disconnected they are. The WCS includes all three factors of the UCLA LS-R: the CTO subscale covers isolation and relational connectedness and collective connectedness is included in CTW.

In a recent large-scale population survey study in the UK, social connection was found to be the strongest protective factor against depression (Choi et al. 2020). The quality and quantity of individuals’ social relationships have been linked not only to mental health but also to both morbidity and mortality; a large meta-analysis found that social relationships are more effective at helping people to live long lives than any other factor, including hypertensive treatment (Holt-Lunstad et al. 2010). Human social genomics has begun to analyse how loneliness affects our immune system and causes chronic inflammation as a precursor to disease (Cole 2014). Combining community development with healthcare has been found to dramatically reduce emergency hospital admissions in a project in the UK where a town was shaped into a ‘compassionate community’ via various interventions whereby people of the town were given the means to connect with each other (Abel 2018).

### Connectedness to world

CTW items represent a state of ‘transpersonal’ ego-transcendence which may be a vital aspect of the therapeutic process catalysed by psychedelics (Pahnke et al. 1970; Roseman et al. 2017; Yaden and Griffiths 2020) and other practices. The CTW subscale relates to ‘self-transcendence’, defined as the capacity to transcend self-boundaries (Reed 1991). The expansion of self-boundaries can occur at the *interpersonal* (opening up one’s sense of self to include other beings) and *transpersonal* (connecting with nature and a spiritual principle) levels (Reed 2008).

All items in the CTW subscale relate to connecting with the world beyond each individual, which is captured also by the item ‘I have felt connected to a purpose in life’. This item was originally hypothesised to belong to the CTS subscale but analyses found it to load onto CTW, suggesting that ‘purpose’ is not related to the good of the individual, but to the good of the world.

CTW contains one item relating to nature connectedness, and one item relating to interconnectedness of everything. Both of these items represent a kind of self-transcendence commonly reported by people after psychedelic experiences, whereby one feels part of an interconnected web of life, and recognises one’s place in the patterns and fabric of the natural world (Noorani et al. 2018). This sense of inter-relatedness is a fundamental aspect of indigenous worldviews from all over the globe. Many indigenous belief systems share a view of people and nature as part of an extended ecological family: for example, the Māori, indigenous people of Aotearoa; the Shipibo of Peru; the Raramuri of Chihuahua, Mexico; and the Skokomish of Washington; the Druids of Wales. These different groups, and many more, separated by geography, culture and time, have described this phenomenon. The Māori worldview (te ao Māori) acknowledges the interconnectedness of all living and non-living things (Rameka 2018). The Raramuri worldview includes ‘Iwigara’: the total interconnectedness of all life, physical and spiritual (Salmón 2000). From the point of view of these belief systems, feeling separate from nature would signify a state of disconnectedness and constitute a significant rupture in wellbeing.

Connectedness to ourselves as part of an inter-related web of life may be essential to the survival of our species (Arce and Winkelman 2021), and this appears to be a common insight occurring during psychedelic therapy, as many quotes from participants in psychedelic research studies attest (Agin-Liebes et al. 2021; Andros Aronovich 2019; Belser et al. 2017; Watts et al. 2017). Such insights are often reported to lead to pro-environmental behaviours (Forstmann and Sagioglou 2017).

If scientific study of connectedness should find that the feeling of being interconnected with nature is associated with wellbeing and pro-environmental behaviour, it will be important to recognise the original, longstanding provenance of this knowledge and include indigenous perspectives in the study of connectedness. For example, the University of Auckland is including the WCS in a study of connectedness and existential distress in Māori and non-Māori people with a life-limiting illness. The project is designed and led by Māori colleagues, who have added two additional CTW items, which reflect Te ao Māori, to supplement the 19-item WCS: ‘I have felt connected to toku whakapapa, my family ancestry’ and ‘I have felt connected to toku whenua, my land’. The dual meaning of ‘whenua’ (‘land’ and ‘placenta’) exemplifies Māori wairua (spirituality), whereby interconnectedness with everything is both grounded and sacred.

The WCS also contains items relating to connecting to a source of universal love and connecting to a spiritual principle, themes which are common in psychedelic therapy, and which again map onto indigenous spiritualities, which can be differentiated from religion and are based on a sense of connectedness and respect for the ‘earth, ancestors, family and peaceful existence’ (Christakis and Harris 2004) ‘an internal connection to the universe’ (Department of Economic and Social Affairs, UN 2009) or an ‘intrinsic, autonomous, and subjective sense of connection with a sacred dimension of reality, which provides meaning, purpose, connection and balance’ (Valentine et al. 2017). The inclusion of spiritual items within the WCS may be considered by some to be antithetical to scientific enquiry. However, as many of the participants in our previous qualitative research (Watts et al. 2017) who indicated spiritual connectedness had been previously non-spiritual, this change in metaphysical beliefs seems like an integral aspect of the psychedelic experience (Timmermann et al. 2021). The importance of mystical-type experience as a predictor of positive outcomes (Roseman et al. 2017; Yaden and Griffiths 2020) requires us to investigate further the relationship between connectedness and spirituality, which the CTW items may facilitate.

### Connectedness as a transdiagnostic factor

Psilocybin showed (statistically significantly) larger change on the WCS than escitalopram. Importantly, the larger change was also statistically significant when looking only at the responders of both groups based on change in depression scores. A triple interaction effect was found between condition (psilocybin vs escitalopram), response (responders vs non-responders) and time (before therapy vs 6-week follow-up). This triple interaction effect supports the hypothesis that the therapeutic mechanism of psilocybin is different from that of escitalopram’s. While both are effective in reducing depression, the clinical response in the psilocybin group was strongly associated with increased connectedness, and less so in the escitalopram group. That there were still increases in WCS scores seen in the escitalopram responders group does not match qualitative reports from patients indicating that SSRI antidepressants can actually lessen a sense of (emotional) connectedness, by making them feel emotionally numbed (Watts et al. 2017). The increases in WCS scores in the escitalopram responders group may be linked to the following: (1) improvements in other domains of connectedness than CTS, as data shows; (2) the extensive personalised care, attention and therapeutic support that participants in both treatment arms of this trial received. In common healthcare practice (in the UK, at least), SSRI medications are administered without extensive psychotherapeutic care; and (3) some non-orthogonality between depression and connectedness.

Psychedelic-assisted therapy is currently applied to different conditions such as depression, anxiety, addiction, anorexia, obesity, chronic pain, OCD and PTSD (Siegel et al. 2021). In case such transdiagnostic application will prove to be effective, a search for an underlying mechanism will become relevant. Alienation, ‘dislocation’ and disconnection have been hypothesised to underlie many mental health conditions, supporting the idea that disconnectedness is a transdiagnostic phenomenon: these include eating disorders (Huemer et al. 2011), borderline personality disorder (Kverme et al. 2019), PTSD (Kearney et al. 2012), depression (Choi et al. 2020; Hari 2019; Karp 2017; Sorajjakool et al. 2008; Watts et al. 2017), addiction (Alexander 2008), anxiety (Taylor et al. 2020) and ADHD (van den Berg and van den Berg 2011). It might be that traumatic experiences in relationships with caregivers, others and society can damage our access to a sense of connectedness and that psychedelic (and other) profound experiences can enable sudden access to that state. This resonates with the reports of people who have had meaningful psychedelic experiences.

To test whether and confirm that the phenomenon of a foundational multidimensional connectedness has validity and clinical utility, it will need to be measured in a range of different clinical populations pre and post ‘treatment’. Future observational and experimental research studies in various settings looking at many different populations are including the WCS in their battery of measures. The CIPPRes Clinic (Central North West London-Imperial Psychopharmacology & Psychedelic Research Clinic) will measure changes in WCS scores for different clinical presentations (i.e. anorexia nervosa, chronic pain, depression, anxiety). This transdiagnostic research avenue will be important for a ‘psychedelicisation’ of medicine, because a successful integration—and not assimilation—of psychedelics into psychiatry may require a change in the way mental and emotional suffering is understood by the dominant paradigms (Schenberg 2018). Alongside transdiagnostic clinical research, psychedelic sessions in group contexts will be a crucial avenue for study, because of the additional boost to connectedness that is experienced in such settings (Kettner et al. 2021).

In the current study, emotional, social and spiritual qualities of the psychedelic experience predicted the changes in connectedness. Psychedelic therapy is intensely context dependent (Hartogsohn 2020)—and thus, the extent to which a participant is able to experience emotional, social and spiritual insights will itself rest on how they are therapeutically prepared for the psychedelic experience, supported during it, and after it. The ‘integration’ phase post dosing is considered essential for acute experiences of connectedness to be consolidated and incorporated into daily life. Integration sessions, where psychedelic therapy has been effective, typically focus more on a person’s wishes for making changes to how they behave within their community and ecosystem than on biomedical issues like changes to their symptoms of depression. Considering connectedness (or its absence) as a transdiagnostically foundational factor is a reminder that—while connectedness can be temporarily boosted by psychedelic therapy—ongoing psychological, communal, political, ecological and spiritual work is needed to maintain connectedness. We believe that the multiple dimensions of connectedness must be addressed by modern models of psychedelic-assisted therapy in order for its safety and efficacy to be optimised. In this way, psychedelics may assist in shifting the dominant biomedical mental health model into a ‘biopsychosocial’ model (Engel 1980; Winkelman 2010), or even a ‘bio-psycho-social-environmental-spiritual’ model, where mental and emotional suffering is not considered as simply a function of individual pathology, but linked to much broader social, environmental and spiritual factors. Interestingly, this model of health maps onto the Shipibo conception of health as encompassing connectedness to self, community and the world (Weiss et al. 2021) and the already well-established model of Māori health, Te Whare Tapa Wha (Rochford 2004), which comprises spiritual, mental, physical and family connectedness.

### Connectedness maintenance post-psychedelic session

One of the biggest challenges facing psychedelic therapy is how to help participants hold on to the benefits which are often lost after a few months (Ortigo 2021). If multidimensional connectedness is one of the main benefits, then being able to study it easily will enable researchers to study different ways of maintaining it after sessions. Without this kind of ‘integration work’, there is a risk that people will become dependent on frequent psychedelic experiences in order to feel connected, rather than developing other practices and contextual changes. It is hoped that the WCS will enable future quantitative measurement of how connectedness typically wanes over time, which may inform the development of therapy protocols with guidance around when repeat sessions could occur. Also, integration approaches for maintaining connectedness, such as community and ecotherapy interventions, can be evaluated and compared.

The WCS may also help identify some of the risks of psychedelic therapy and could offer particular usefulness in helping us learn more about some commonly experienced difficulties in the much understudied integration phase (post-psychedelic therapy), as clinical trials only collect data for a few months after the session. Sometimes, the integration period can be a time of confusion and overwhelm. Therapists working with people who have had psychedelic sessions and need further support (integration therapists) tend to advise staying present in the body, senses and emotions in the weeks after a psychedelic session. Embodied self-awareness and connection to a supportive and understanding community are both essential to psychedelic integration, in order to ‘ground’ a profound and potentially destabilising connection to new ideas. Whereas connection to world can be thought of as the branches of a tree reaching up to the sky, connection to self can be considered the trunk of the tree, and connection to others the root system.

Thus, ‘over-connecting’ to the world (CTW) without a solid foundation of connection to self (CTS) could indicate the need for psychotherapeutic integration and grounding, rather than further psychedelic sessions. Some examples of over-connecting to CTW without a strong CTS could be an ‘ontological shock’ (Davis et al. 2020b; Timmermann et al. 2021)—whereby assumptions about what it is to be a conscious living being on earth are deeply challenged—which can sometimes precipitate hypo-manic or manic reactions (Hendin and Penn 2021), ‘spiritual bypassing’ (Masters 2010) and ‘spiritual narcissism’ (Lasch 1979/2018; 1987).

Another risk of the integration period of psychedelic therapy is the experience of intense disappointment. For individuals who have felt disconnected for much of their life, a sudden burst of connectedness that lasts for a few months but then dwindles may be a risk factor in self-harm or suicide. Therefore, measuring an individuals’ connectedness to self, others and the world in the months after a psychedelic session may be important for safety,

## Limitations

The current validation of WCS has a number of limitations:

1. The validation of the WCS is limited by a self-selection bias for the online questionnaire, and potentially also the RCT—e.g., where the majority of volunteers were psilocybin therapy preferring (vs. the SSRI treatment). Pop culture surrounding psychedelics tends to feature elements of connectedness and it is possible that survey and RCT participants were familiar with related topics and how psychedelics may enhance them. Study participants were predominantly WEIRD (white, educated, industrialised, rich, democratic) (Henrich et al. 2010) reflecting a pervasive problem in psychedelic research (George et al. 2020). Future studies should test the scale in the wider population.
2. Many participants dropped out of the survey study, which could have created attrition bias effects; e.g., those who did not benefit from their psychedelic experience may have been more likely to discontinue. Future studies should take measures to solve such problems, possibly by introducing more incentives for completion of the study.
3. Comparison with more questionnaires are required to further test the convergent and discriminant validity of the WCS.
4. Following the advice by Jackson et al. (2014) on multiple publications from the same study sample, the following publications should be noted, which are based on the same survey study samples used for internal validation of the WCS: Kettner et al. (2021) researched group experiences of psychedelic *communitas*; Timmermann et al. (2021) studied changes in metaphysical beliefs; Zeifman et al. (2020) researched experiential avoidance; Kuc et al. (2021) researched the interaction of psychedelics with cannabis; and Spriggs et al. (2021) researched changes in wellbeing in individuals with eating disorders. Importantly, however, these publications explored clearly distinct research questions from the present paper.
5. The item creation process for the WCS was based on qualitative reports from a depression study and may therefore be biased by this sample—and by the qualitative nature of that original analysis. In order to assess whether the final validated measure was considered an accurate and appropriate tool by those who had themselves experienced a psychedelic-assisted therapy–induced connectedness, the WCS was submitted to the Psychedelic Participant Advisory Network, (PsyPAN) who were asked to review it and consider whether there were any aspects of psychedelic therapy–induced connectedness that were not represented by the measure, or any existent items which they thought did not belong there or were disagreed with. PsyPAN’s feedback was that the measure could have asked about connecting to personal values as part of ‘connectedness to world’, for example, becoming vegetarian, changing to a more meaningful job or joining activist networks. An item relating to ‘connectedness to values’ was in fact in the original 23-item WCS in the CTS scale but removed after factor analysis because it loaded onto both connectedness to self and connectedness to world. Two other items were removed for the same reason: ‘I have felt connected to deeper aspects of myself’ and ‘I have felt connected to insight/intuition’. These three items taken together point perhaps to a type of self-connectedness which has not been included in the final WCS, which could be conceptualized as less changeable than embodied, sensory and emotional experience, which tends to be transitory. A ‘deeper’ more immutable aspect of self may need to be re-introduced to measures of connectedness, despite its crossloading here on both CTS and CTW. This crossloading in itself is worthy of future study.
6. The 19-item measure is not exhaustive. There will be aspects of connectedness that are not included in the current WCS. This 19-item measure is a starting point. It is hoped that others will suggest items that are relevant based on their observations. It would be possible to have different versions of this scale validated for different populations, perhaps with the ‘W’ removed and replaced with a more appropriate letter for that context. Until that time, we hope the current validated WCS will be a starting point for the quantitative study of the state of feeling connected to self, others and the world.

## Conclusions

Previous findings that many different dimensions of connectedness are linked to positive mental health outcomes suggest that connectedness, in all its forms, should be carefully explored and defined. The present work supports the view that a fundamental multidimensional state of connectedness exists and has developed a scale that defines and measures this phenomenon. Previously, research findings regarding connectedness have tended to probe only sub-dimensions of the phenomena, making it difficult to compare results and build the research corpus. Having a single measure comprising multiple dimensions of connectedness may help to consolidate the view that these sub-dimensions are inter-related and underpinned by a common generalised connectedness. If used widely, the WCS may help us understand how different aspects of connectedness relate to each other and the interventions and conditions under which connectedness is experienced and maintained. Psychedelic-assisted therapy may prove to be one of the most effective interventions for increasing connectedness among individuals and groups of people—and future research may reveal how important this effect is for improving and maintaining mental health.

## Supplementary Information

Below is the link to the electronic supplementary material.

Supplementary file1 (PDF 297 KB)

## Footnotes

1 Note that we regard the vernacular terms ‘connection’ and ‘disconnection’ as synonymous with ‘connectedness’ and ‘disconnectedness’, where the former can operate as verbs and the latter as nouns.
