# Supplementary material for: The Watts Connectedness Scale: a new scale for measuring a sense of connectedness to self, others, and world
Source: Psychopharmacology (Berl). 2022 Aug 8;239(11):3461–83. doi: 10.1007/s00213-022-06187-5 (PMC9358368; doi:10.1007/s00213-022-06187-5)
Supplement: Supplementary file 1 — Supplementary file1 (PDF 297 KB) [file 213_2022_6187_MOESM1_ESM.pdf]

The Watts Connectedness Scale: a new scale for measuring a sense of connectedness to self, others, and world.

Psychopharmacology

Supplementary Material

Rosalind Watts[1,2,3,*], Hannes Kettner[1], Dana Geerts[1,4], Sam Gandy[1], Laura Kartner[1], Lea Mertens[1], Christopher Timmermann[1], Matthew M Nour[1], Mendel Kaelen[1], David Nutt[1], Robin Carhart-Harris[1,5], Leor Roseman[1*]

[1]Centre for Psychedelic Research, Imperial College London, London, United Kingdom
[2]Acer Integration, London, United Kingdom
[3]Synthesis Institute, Amsterdam, Netherlands
[4]Department of Neuropsychology and Psychopharmacology, Faculty of Psychology and Neuroscience, Maastricht University, Maastricht, Netherlands
[5]Psychedelics Division, Neuroscape, Department of Neurology, University of California San Francisco, USA

*Communicating authors:
Rosalind Watts
drwatts@synthesisretreat.com

Leor Roseman
leor.roseman13@imperial.ac.uk

Table S1. Exploratory factor analysis factor loadings in the Global Psychedelic Survey dataset.

Item	CTW	CTS	CTO
*I have felt trapped in my mind.**	0.071	-0.185	**0.533**
My mind has felt connected to my heart/emotion.	0.021	**0.667**	-0.065
I have felt connected to my senses (touch, taste, sight smell, hearing).	0.072	**0.559**	-0.088
I have felt connected to a range of emotions.	-0.074	**0.839**	-0.009
If I had chosen to, I could have 'sat with' painful memories.	0.082	**0.319**	0.051
I have felt connected to my body.	0.064	**0.594**	-0.138
I have felt connected to deeper aspects of myself.	0.447	0.454	-0.016
I have felt connected to insight/intuition.	0.467	0.404	0.036
I have felt connected to my values.	0.299	0.484	-0.006
I have been able to fully experience emotion, whether positive or negative.	-0.017	**0.706**	-0.009
*I have felt alone.**	0.003	0.011	**0.749**
I have felt connected to friends and/or family.	0.120	0.244	**-0.528**
I have felt connected to a community.	0.261	0.063	**-0.536**
I have felt connected to strangers.	0.523	-0.103	-0.335
I have felt connected to all humanity.	**0.684**	-0.051	-0.235
*I have felt unwelcome amongst others.**	0.090	0.015	**0.616**
*I have felt separate from the world around me.**	0.036	-0.016	**0.727**
I have felt connected to a purpose in life.	**0.519**	0.216	-0.079
I have felt connected to nature.	**0.642**	0.084	-0.018
I have felt connected to a spiritual essence (in the secular or religious sense).	**0.747**	-0.055	0.024
I have felt connected to a source of universal love.	**0.813**	-0.022	-0.045
I have seen things from a broad perspective, 'the bigger picture'.	**0.753**	0.068	0.100
I have felt that everything is interconnected.	**0.776**	0.046	0.101

Oblimin rotation. CTS: Connectedness to Self, CTO: Connectedness to Others, CTW: Connectedness to World.

*negatively worded items are reverse coded

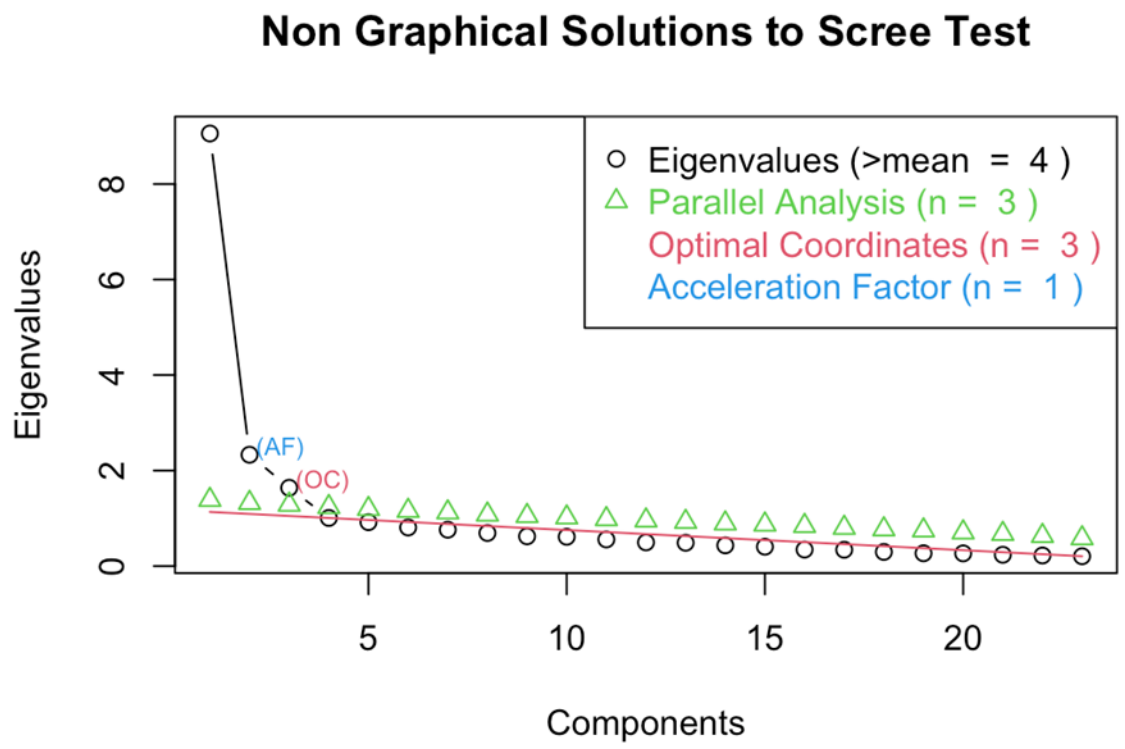

Figure S1. Scree plot showing eigenvalues and results of non-graphical tests to determine optimal number of factors to extract from the Watts' Connectedness Scale.

Watts' Connectedness Scale (WCS)

<u>Reference</u>

Watts R, Kettner H, Geerts D, Gandy S, Kartner L, Mertens L, Timmermann C, Nour M, Kaelen M, Carhart-Harris R, Roseman L (2022). The Watts Connectedness Scale: a new scale for measuring a sense of connectedness to self, others, and world. *Psychopharmacology*

<u>Instructions</u>

Reflecting on how you have felt over the past 2 weeks, please rate the following items on a scale from **'Not at all' to 'Entirely'** according to how you have felt over this time period. Please answer every item, even if you are unsure or feel the item is unclear or poorly worded. Drag the indicator to a position on the scale that shows how much you agree or disagree with each of the following statements.

<u>Response format</u>

Each item is rated on a 0 – 100 visual analogue scale with the anchors 0 = Not at all, 100 = Entirely

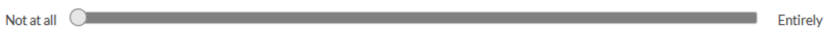

<u>Final items</u>

1. *I have felt trapped in my mind.*
2. *My mind has felt connected to my heart/emotion.*
3. *I have felt connected to my senses (touch, taste, sight smell, hearing).*
4. *I have felt connected to a range of emotions.*
5. *If I had chosen to, I could have 'sat with' painful memories.*
6. *I have felt connected to my body.*
7. *I have been able to fully experience emotion, whether positive or negative.*
8. *I have felt alone.*
9. *I have felt connected to friends and/or family.*
10. *I have felt connected to a community.*
11. *I have felt connected to all humanity.*
12. *I have felt unwelcome amongst others.*
13. *I have felt separate from the world around me.*
14. *I have felt connected to a purpose in life.*
15. *I have felt connected to nature.*
16. *I have felt connected to a spiritual essence (in the secular or religious sense).*
17. *I have felt connected to a source of universal love.*
18. *I have seen things from a broad perspective, 'the bigger picture'.*
19. *I have felt that everything is interconnected.*

Scoring

Connectedness to Self (CTS): (WCS2 + WCS3 + WCS4 + WCS5 + WCS6 + WCS7) / 6

Connectedness to Others (CTO): ((100 – WCS1) + (100 – WCS8) + WCS9 + WCS10 + (100 – WCS12) + (100 – WCS13)) / 6

Connectedness to World (CTW): (WCS11 + WCS14 + WCS15 + WCS16 + WCS17 + WCS18 + WCS19) / 7

General Connectedness (WCS): (CTS + CTO + CTW) / 3
